# ﻿Special article—EchoPeer: a standardized framework for assessing echocardiography reports in the era of artificial intelligence: a recommendation from the Korean Society of Echocardiography AI and Future Strategy Committee

**DOI:** 10.1186/s44348-026-00085-6

**Published:** 2026-08-14

**Authors:** SungA Bae, Inki Moon, Jiesuck Park, Jaehyun Lim, Seung-Ah Lee, Yeonyee E. Yoon, Goo-Yeong Cho

**Affiliations:** 1 Division of Cardiology, Department of Internal Medicine, Yongin Severance Hospital, Yonsei University College of Medicine, Yongin, Republic of Korea; 2 Division of Cardiology, Department of Internal Medicine, Soonchunhyang University Bucheon Hospital, Soonchunhyang University College of Medicine, Bucheon, Republic of Korea; 3 Cardiovascular Center and Division of Cardiology, Department of Internal Medicine, Seoul National University Bundang Hospital, Seongnam, Republic of Korea; 4 Division of Cardiology, Department of Internal Medicine, Seoul National University Hospital, Seoul National University College of Medicine, Seoul, Republic of Korea; 5 Ontact Health, Seoul, Republic of Korea

**Keywords:** Echocardiography, Artificial intelligence, Reproducibility of results, Diagnostic errors, Patient safety

## Abstract

Artificial intelligence (AI) is rapidly advancing from automated measurement to full-report generation, yet existing frameworks do not provide a unified scoring approach for both human- and AI-authored reports. We developed EchoPeer, a three-step evaluation framework for echocardiography reports, scored against a reference standard. Step 0 (safety score, %) is a pass/fail safety gate for life-threatening findings (critical omission and hallucination). Step 1 (precision score, 0–80 scale) scores per-item diagnostic accuracy across 25 items in four anatomical domains. Step 2 (quality score, 0–20 scale) grades clinical utility across three checkpoints: relevance, synthesis, and clarity. The precision and quality scores are summed as a composite score (0–100 scale), with step 0 failures scored as 0. EchoPeer was applied to 30 adult transthoracic echocardiography cases interpreted by 11 human readers across three training levels. All three EchoPeer scores increased monotonically with training level. Median safety scores were 86.7%, 90.0%, and 96.7% for levels 1, 2, and 3, respectively (P = 0.005), and sensitivity for critical finding detection rose from 59.3% to 92.6% (P = 0.007). Median precision scores were 61.8, 64.7, and 70.9, respectively (P = 0.007), and median quality scores were 10.4, 12.9, and 15.1, respectively (P = 0.009). The composite score followed the same gradient (72.2, 77.7, and 86.2, respectively; P = 0.003), and the precision and quality scores correlated significantly at both the case level (Spearman ρ = 0.568) and the participant level (ρ = 0.888). EchoPeer discriminates clinical competence in human readers and produces an error profile that mirrors known challenges in echocardiographic reporting, providing a clinically grounded foundation for the future evaluation of AI-generated echocardiography reports.

**Supplementary Information:**

The online version contains supplementary material available at 10.1186/s44348-026-00085-6.

## Background

Echocardiography is the most widely used cardiac imaging modality, and its reports drive clinical decision-making. Over the past 5 years, artificial intelligence (AI) in echocardiography has advanced from single-task models for ejection fraction estimation and view classification [1] to multimodal foundation models trained on large-scale paired video–report datasets that can draft entire study interpretations [2–4]. Vision-language systems such as EchoCLIP, PanEcho, and EchoPrime now produce free-text descriptions of cardiac chambers, valves, and pericardium, generating echocardiography reports authored entirely by AI rather than isolated measurement alone. The technological capacity to generate such reports has progressed faster than the clinical infrastructure needed to validate them [5].

Generative AI introduces error modes that traditional measurement-validation studies do not capture. Large language models (LLMs) can produce content that reads as authoritative while containing fabricated findings or critical omissions [6, 7]. Hallucinated diagnoses have been documented in multimodal AI systems applied to radiology [8], and in open-source LLMs applied to echocardiographic report summarization, where fabricated chamber sizes and Doppler measurements have been reported [9]. Current evaluations of medical AI often rely on multiple-choice benchmarks or accuracy on isolated tasks. While such designs may demonstrate apparent equivalence to clinicians, they do not capture the safety failures that emerge when an AI system produces a full, unstructured clinical report, where errors of synthesis and omission, not just measurement, must be evaluated [10]. For echocardiography, in which a missed diagnosis, such as pulmonary embolism, tamponade, or aortic dissection directly affects survival, clinical hallucination and critical omission must be screened before any downstream accuracy metric can be meaningfully interpreted.

Existing evaluation tools were not designed for this task (Table 1) [11–15]. Radiologist peer review programs such as RADPEER were built for inter-reader agreement among human readers and do not separate fabrication from interpretive variance [11–13]. Training-based competency framework (the COCATS 4 Task Force 5 statement) defines skill tiers for human readers and is not directly applied to algorithmic systems [14]. The 2025 American Society of Echocardiography (ASE) Guidelines for the Standardization of Adult Echocardiography Reporting specify the expected content of an adult echocardiography report but provide a descriptive rather than evaluative framework, without a scoring rubric for comparing reports against a reference standard [15]. None of these tools include an explicit safety gate that screens out reports with critical fabrication or omission before accuracy is scored.

**Table 1:** Comparison of existing evaluation frameworks for echocardiography reporting

Attribute	RADPEER [11–13]	COCATS 4 Task Force 5 [14]	2025 ASE Guidelines [15]	EchoPeer (present study)
Primary purpose	Peer review for inter-reader agreement	Definition of training tiers for human readers	Specification of expected report content	Structured evaluation of AI and human reports against a reference standard
Primary target	Human-authored radiology reports	Cardiology fellows and practicing physicians	Adult TTE reports (any author)	AI-generated and human-authored adult TTE reports
Patient safety domain	Implicit; embedded within discrepancy grading	Not addressed	Not addressed	Explicit step 0 fail-switch for 7 critical findings
Diagnostic accuracy scoring	4-Tier discrepancy scale	Not provided	Not provided	Per-item weighted scoring across 25 items, 4 domains (precision score, 0–80 scale)
Hallucination/fabrication detection	Not specifically defined	Not addressed	Not addressed	Defined at step 0 (critical hallucination) and step 1 (major error)
Applicability to AI systems	Not designed for AI	Not portable to algorithms	Descriptive only (content specification only)	Designed from the evaluation of both AI-generated and human-authored reports on a common scale
Output metrics	Single discrepancy grade	Tier classification (I, II, III)	None (qualitative recommendations only)	Safety score (%), precision score (0–80 scale), quality score (0–20 scale), and composite score (0–100 scale)

AI, artificial intelligence; ASE, American Society of Echocardiography; COCATS, Core Cardiovascular Training Statement; RADPEER, Radiology Peer Review program of the American College of Radiology; TTE, transthoracic echocardiography

To address this gap, the Korean Society of Echocardiography established the AI and Future Strategy Committee to develop a standardized assessment framework for echocardiography reports. The framework, designated EchoPeer, was designed from inception to be applicable to both AI-generated and clinician-authored reports scored against a reference standard. EchoPeer comprises three sequential steps: (1) a safety gate that screens each report for critical findings whose omission and fabrication would affect patient safety; (2) a precision assessment that quantifies per-item diagnostic accuracy across 25 items in four anatomical domains; and (3) a quality assessment that grades clinical utility through three checkpoints: relevance, synthesis, and clarity.

A framework intended for AI evaluation must first demonstrate that it can distinguish meaningful differences in report quality among human readers of varying experience. Here, we present the EchoPeer protocol and report its pilot validation in cardiology fellows and junior staff members across three training levels, examining whether the three EchoPeer scores capture the gradient of clinical competency expected from training level. This step establishes construct validity in human readers as a prerequisite for the planned subsequent application of EchoPeer to AI-generated reports.

## EchoPeer assessment protocol

**Fig. 1:**
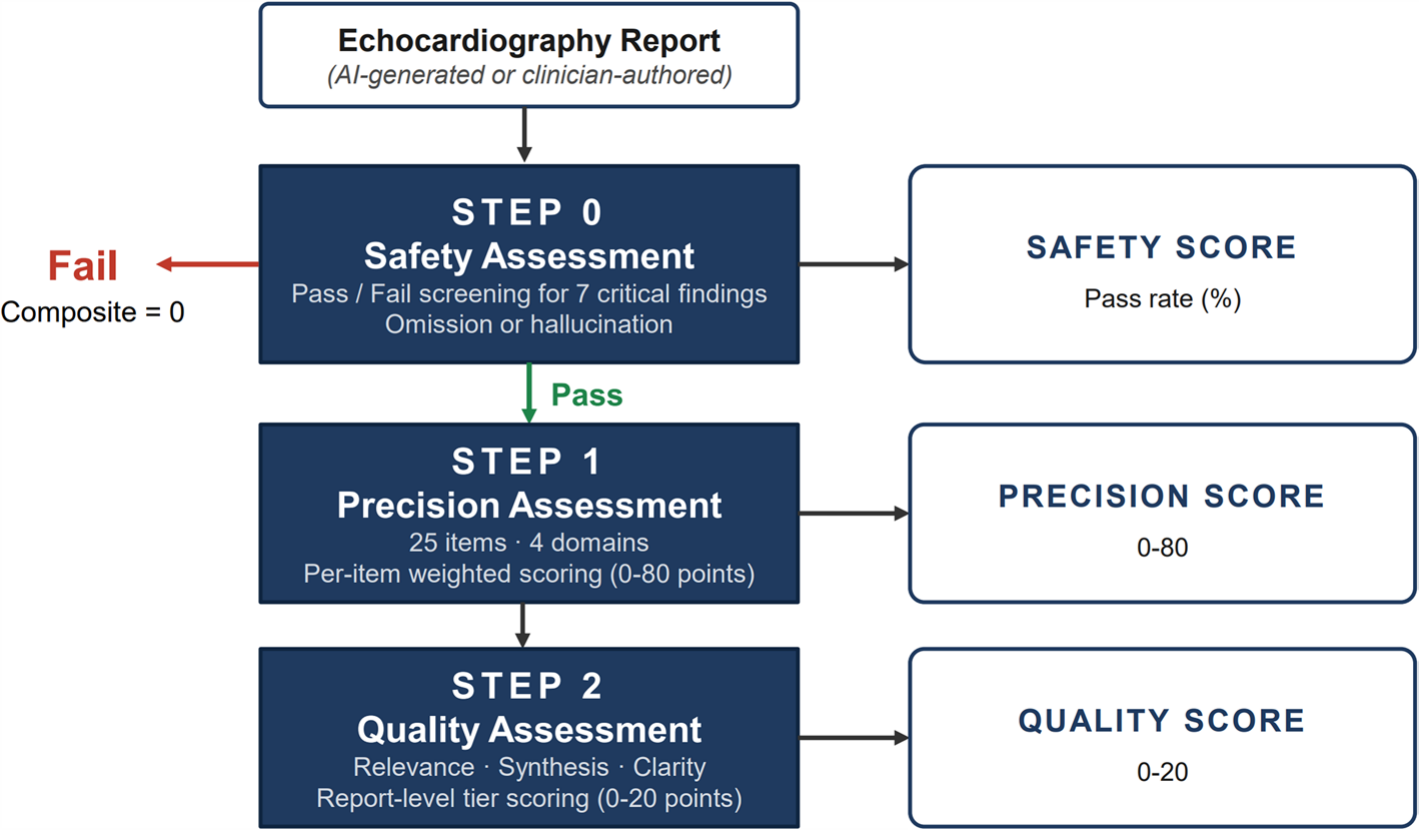
EchoPeer assessment workflow. Three sequential assessment steps (step 0, safety; step 1, precision; step 2, quality) generate three independent scores. Reports failing step 0 are assigned step 1 = 0, step 2 = 0, and consequently composite score = 0 in the analysis

The EchoPeer protocol is a structured framework for evaluating AI-generated and clinician-authored adult transthoracic echocardiography reports against a predefined consensus reference standard. The protocol comprises three sequential steps (Fig. 1). Step 0 yields a safety score (%) representing the proportion of cases passing step 0; this score serves as an independent safety gate. Step 1 yields a precision score (0–80 scale), and step 2 yields a quality score (0–20 scale); these two scores are summed at the case level to form the EchoPeer composite score (0–100 scale), which represents the integrated diagnostic accuracy and clinical utility of the report. A case failing step 0 receives a composite score of 0 points (precision = 0, quality = 0), reflecting the clinical principles that failure to identify a critical finding renders the report clinically unacceptable regardless of remaining accuracy. EchoPeer is designed for report level scoring; reader-level performance is derived by aggregation across each reader’s cases. The framework is reference standard–agnostic, allowing investigators to anchor scoring to expert consensus, core laboratory interpretation, or preexisting clinical reports depending on study design and scale. The framework itself is intended to remain stable across versions, while the specific clinical guidelines used to define reference categories and grading thresholds within each step are external to the framework and updated as the evidence base evolves.

Step 0 (safety assessment) screens each report for critical errors that directly affect patient safety (Table 2). The step 0 architecture—pass/fail screening with critical omission and critical hallucination as the two failure modes—is a stable component of the framework. The specific list of critical findings in the current framework version was derived from the 2025 ASE guidelines for the Standardization of Adult Echocardiography Reporting [15] and may be revised in future versions as evidence and guidelines evolve. Seven life-threatening findings evaluated in the present framework are: (1) suspected cardiac tamponade; (2) suspected aortic dissection or acute aortic syndrome (AAS); (3) complication of myocardial infarction (MI), including ventricular septal rupture, ventricular or papillary muscle rupture, or pseudoaneurysm; (4) thrombus in transit; (5) acute right ventricular (RV) dysfunction and suspected acute pulmonary embolism (PE); (6) left ventricular (LV) assist device or venoarterial extracorporeal membrane oxygenation complications; and (7) severe valve obstruction or stenosis in prosthetic or native valves, especially if acute or new.

**Table 2:** EchoPeer step 0 safety assessment: critical findings (summary)

No	Critical finding	Clinical definition (brief)
1	Suspected cardiac tamponade	Tamponade physiology with chamber collapse and IVC plethora
2	Suspected aortic dissection or acute aortic syndrome	Aortic dissection, intramural hematoma, or penetrating ulcer
3	Complications of myocardial infarction	Ventricular septal rupture, ventricular or papillary muscle rupture, or pseudoaneurysm
4	Thrombus in transit	Mobile right-sided thrombus with high embolism risk
5	Acute RV dysfunction and suspected acute PE	Acute RV pressure overload pattern suggesting acute PE
6	LV assist device or VA-ECMO	Acute LVAD or VA-ECMO complication requiring immediate management
7	Severe valve obstruction/stenosis in prosthetic or native valves	Severe stenosis or obstruction of any cause, especially if acute or new

Critical findings were adopted from Table 14 of the 2025 ASE Guidelines for the Standardization of Adult Echocardiography Reporting [15] and may be revised in future framework versions as evidence and guidelines evolve. The step 0 architecture—pass/fail screening with critical omission and critical hallucination as the two failure modes—remains stable across versions. For each critical finding, two failure modes are scored: critical omission (reference-positive but unmentioned or denied) and critical hallucination (reference-negative but asserted as present or probable). Reports that explicitly mark uncertainty are not classified as failures. Detailed clinical definitions and full omission and hallucination fail criteria are provided in Table S1

IVC, inferior vena cava; LV, left ventricle; LVAD, left ventricular assist device; PE, pulmonary embolism; RV, right ventricle; VA-ECMO, venoarterial extracorporeal membrane oxygenation

For each finding, two failure modes are scored. Critical omission is defined as a reference-positive finding that is unmentioned, denied, or definitively downgraded to a noncritical category in the report; a report that preserves the critical category (e.g., “moderate-to-severe aortic stenosis” when reference is severe aortic stenosis) is not classified as failure. Critical hallucination is defined as a reference-negative finding asserted in the report as definitely present; reports that explicitly mark uncertainty (e.g., “possible cardiac tamponade, cannot be excluded”) are not classified as failures. A case is classified as “fail” if any of the seven findings is failed for that case; otherwise, the case passes step 0. The safety score for a reader is the proportion of cases passing step 0. Cases failing step 0 are assigned a precision score of 0 and quality score of 0, and are included in the reader’s mean score rather than excluded, so that failure to detect critical finding is reflected in overall performance. Detailed critical finding definitions and full omission and hallucination fail criteria are provided in Table S1.

Because step 0 is intended as a safety gate, a step 0 failure is treated differently from an ordinary item-level error. A report that omits a predefined life-threatening finding or introduces a false-positive critical finding cannot be considered clinically safe, even if other noncritical components are accurate. Assigning a nonzero composite score in such cases could allow a report with a critical safety failure to appear acceptable because of preserved performance in lower-risk domains. Therefore, when step 0 fails, both the precision score and quality score are set to 0, resulting in a composite score of 0, and the failed case is retained in the reader’s mean score rather than excluded. This rule preserves the safety-first purpose of EchoPeer and ensures that critical finding failure is not diluted by otherwise accurate report content.

Step 1 (precision assessment) quantifies per-item diagnostic accuracy through a weighted scoring approach. The step 1 architecture, a four-tier accuracy scale applied to items grouped by anatomical domain, with item-level weights aggregated across domains into a single precision score, is a stable component of the framework. Each item is assigned a weight (W) reflecting its clinical importance, and domain-level weighting arises from the summed weights of the items within each domain. The evaluated domains and items were anchored to the core reporting elements recommended in the 2025 ASE reporting standards and disease-specific echocardiography guidelines [16–19]. However, these guidelines do not provide a validated numerical weighting scheme for report-evaluation domains or items. Therefore, the current weights represent an initial expert-derived operational scale based on clinical impact, patient safety relevance, and expected influence on management, and should be recalibrated in future validation studies. In the current framework version, 25 items are evaluated across four anatomical domains: domain A, left heart structure and function (seven items, 22 points); domain B, valves (nine items, 28 points); domain C, right heart and pulmonary artery (four items, 14 points); and domain D, aorta, pericardium, intracardiac mass, and congenital heart disease (five items, 16 points), totaling 80 points (Table 3). The relative weight assigned to each domain reflects expert judgment of its clinical impact in routine echocardiographic reporting. Pulmonary valve disease and extracardiac findings are excluded from step 1 scoring in the current framework version because of sparse standardized grading criteria.

**Table 3:** EchoPeer step 1 precision assessment: domain structure

Domain	Representative item	No. of items	Weight (points)
A (left heart structure and function)	LV cavity size; LV mass and geometry; LVEF; RWMA presence and severity; LA size; LV diastolic function	7	22
B (valves)	Aortic valve morphology, and stenosis, and regurgitation severity; mitral valve morphology, and stenosis, and regurgitation severity; tricuspid regurgitation severity; prosthetic valve presence, type, location, and dysfunction	9	28
C (right heart and pulmonary artery)	RV size and structure; RV systolic function; pulmonary hypertension; IVC and right atrial pressure	4	14
D (aorta, pericardium, mass, and CHD)	Aorta (per segment); pericardium; intracardiac mass; simple and complex congenital heart disease	5	16
Total		25	80

Each item is graded on a four-tier scale, with the score determined by the assigned weight (W): “exact” (W × 1.00), “acceptable” (W × 0.75), “discrepancy” (W × 0.50), and “major error” (W × 0.25). Items for which the reference is missing, or the item is excluded by protocol are designated NA and excluded from the calculation. Item weights reflect the clinical importance of each item, and domain weights reflect the relative clinical impact of findings within each anatomical region, both assigned by expert consensus. The precision score is the weighted sum of tier scores across evaluable items, rescaled to the total weight of those items and expressed on a 0–80 scale. The complete 25-item table with reference categories and item-level scoring principles, together with the operational scoring rules, is provided in Tables S2 and S3

CHD, congenital heart disease; IVC, inferior vena cava; LA, left atrium; LV, left ventricle; LVEF, left ventricular ejection fraction; NA, not applicable; RV, right ventricle; RWMA, regional wall motion abnormality

Each item is graded against the reference on a four-tier scale: “exact” (W × 1.00) when the report matches the reference on the relevant grade or category; “acceptable” (W × 0.75) when the report differs by one adjacent grade without altering clinical management; “discrepancy” (W × 0.50) when the report differs by two grades at a level that may change management; or “major error” (W × 0.25) when the report differs by three or more grades, involves direction-reversal errors, asserts a false-positive major finding, or actively denies a reference-positive major finding. A residual fraction of the weight is retained even at the major error tier, so that the score reflects graded severity rather than a binary pass/fail at the item level. Items are scored as not applicable (NA) when the reference is missing, or the item is excluded by protocol. Items with internal inconsistency within a single report are scored under the worst-direction normalization rule.

The precision score is the weighted sum of tier scores across evaluable items, rescaled to the total weight of those items and expressed on a 0–80 scale; NA items are excluded from both the numerator and this rescaling denominator, so that omitted items neither penalize nor inflate the score. The operational scoring rules governing these components, including the adjacent-grade parameters, implicit normal categories, indeterminate handling, item-specific omission rules, internal inconsistency protocols, and NA categories, are provided in Table S2, and the complete 25-item assessment template, featuring specific weights, reference categories, and item-level scoring nuances, is provided in Table S3.

Step 2 (quality assessment) grades the clinical utility of each report on a four-tier scale: optimal (20 points), adequate (16 points), suboptimal (10 points), and unacceptable (0 points) (Table 4). The step 2 architecture is a stable component of the framework, applying this four-tier scale through three checkpoints: relevance, synthesis, and clarity. The four-tier graded structure was inspired by the RADPEER peer review program, which grades interpretive discrepancies on ordinal scale of increasing clinical significance [13]; the three checkpoints, however, were developed specifically for echocardiographic report assessment. The point values assigned to the four step 2 tiers were chosen to map this ordinal assessment onto the 20-point quality score domain of EchoPeer: 20 points for full quality credit, 16 points for reports that remain clinically usable despite minor limitations, 10 points for reports with clinically meaningful reduction in usefulness, and 0 points for failure of report-level clinical utility. These thresholds represent an initial expert-derived operational scale for the present framework and will require further calibration in larger validation studies. Relevance assesses whether the report directly answers the clinical question posed by the indication; synthesis assesses whether the findings are logically integrated without internal contradictions; and clarity assesses whether the report is concise and complete enough for downstream decision-making. These checkpoints reflect dimensions of report quality emphasized in structured reporting initiatives and echocardiographic laboratory quality framework [20, 21]. When the reference establishes a cardiomyopathy phenotype (hypertrophic cardiomyopathy, dilated cardiomyopathy [DCM], restrictive cardiomyopathy, arrhythmogenic RV cardiomyopathy, or nondilated LV cardiomyopathy) [16], an additional expectation is applied within the relevance and synthesis checkpoints: the report should frame the phenotype with appropriate uncertainty and supporting evidence. The quality score is assigned at the report level by holistic judgment across the three checkpoints, following the tier definitions detailed in Table 4. The precision score (0–80 scale) and the quality score (0–20 scale) are summed at the report level to form the EchoPeer composite score (0–100 scale), integrating per-item diagnostic accuracy with overall clinical utility.

**Table 4:** EchoPeer step 2 quality assessment rubric

Grade	Score	Summary criteria	Cardiomyopathy phenotype trigger application
Optimal	20	Ready for immediate clinical use Conclusions are clear and well supported by evidence; the report fully provides the information required for clinical decisions	Phenotype is named and appropriately framed, with clear evidence-conclusion linkage. A next step recommendation is not required for optimal when core findings are complete
Adequate	16	Acceptable for clinical use without modification Core conclusions are preserved; minor omissions or ambiguities are present but do not affect clinical decisions	Phenotype framing may be somewhat generic or evidence linkage may be incomplete, but the central interpretation is preserved without clinical misinterpretation
Suboptimal	10	Requires modification before clinical use Conclusions are ambiguous, evidence is insufficient, or synthesis is incomplete in ways that may alter management	Phenotype framing is missing when clinically expected, evidence linkage is insufficient, or phenotype selection is inaccurate (without rising to a misleading definitive assertion)
Unacceptable	0	Not acceptable for clinical use Substantial internal contradictions, mismatched evidence and conclusions, or extensive omission or fabrication of central findings	Major phenotype (e.g., HCM, ARVC, amyloidosis) is asserted definitively without evidence, or the conclusion contradicts strong reference findings, producing critical clinical misinterpretation

Quality assessment uses a four-tier rating scale adapted from the RADPEER peer review framework [13]. A single report-level grade is assigned based on holistic judgment across three checkpoints: relevance (does the report directly answer the clinical question?), synthesis (are findings logically integrated without internal contradiction?), and clarity (is the report concise and complete enough for downstream decision-making?). The cardiomyopathy phenotype trigger applies when the reference establishes HCM, DCM, RCM, ARVC, or NDLVC, and additional phenotype framing expectations are applied within relevance and synthesis checkpoints. Cases failing step 0 are assigned a quality score of 0 and are retained in the step 2 denominator

ARVC, arrhythmogenic right ventricular cardiomyopathy; DCM, dilated cardiomyopathy; HCM, hypertrophic cardiomyopathy; NDLVC, nondilated left ventricular cardiomyopathy; RCM, restrictive cardiomyopathy

## Pilot validation: feasibility and discriminative performance

### Ethics statement

The protocol was approved by the Institutional Review Board of Seoul National University Bundang Hospital (No. B-2602–1026-301). All participants provided written informed consent prior to participation.

### Study participants

Eleven echocardiography fellows and junior staff members from multiple training hospitals participated in the pilot evaluation. Training levels were assigned by a COCATS 4–derived self-assessment questionnaire that incorporated echocardiography rotation duration and lifetime case volume (transthoracic echocardiography [TTE] and transesophageal echocardiography [TEE], stress echocardiography, 3D echocardiography, and strain imaging) (Appendix S1) [17, 18]. Participants were classified into three training levels: level 1 (n = 3; first-year fellows with limited echocardiographic interpretation experience), level 2 (n = 5; second-year or early third-year fellows), and level 3 (n = 3; staff members within 2 years of completing fellowship training and actively involved in echocardiographic interpretation). Echocardiography experience differed significantly across levels for training year (P = 0.002), echocardiography rotation duration (P = 0.012), TTE interpretation volume (P = 0.044), stress echocardiography volume (P = 0.028), and strain imaging volume (P = 0.044). Baseline characteristics are summarized in Table 5.

**Table 5:** Baseline characteristics of participants

Characteristic	Level 1 (n = 3)	Level 2 (n = 5)	Level 3 (n = 3)	P-value
Training year (median)	1	2	≥ 3	0.002
Echocardiography rotation (mo)	4 (1–5)	6 (6–10)	36 (13–60)	0.012
TTE interpreted (median category)	50–149	≥ 750	≥ 750	0.044
Stress echocardiography (median)	0	5	50	0.028
Strain imaging (median)	5	10	200	0.044

Values are presented as median (range) or median category number (%). Training levels were assigned by a COCATS 4–derived self-assessment questionnaire (Appendix S1) incorporating echocardiography rotation duration, lifetime case volume, self-assessment, and supervisor evaluation. P-values were calculated by Fisher exact test (training year) or the Kruskal–Wallis test (continuous and ordinal variables)

TTE, transthoracic echocardiography

### Study design

Thirty representative adult TTE cases were selected to represent major clinical categories, including normal studies, ischemic heart disease, valvular heart disease, various cardiomyopathies, acute PE, AAS, and simple and complex congenital heart disease. All cases were deidentified prior to distribution. Cases were provided to participants in DICOM format and reviewed through a clinical picture archiving and communication system (PACS) environment (INFINITT, INFINITT Healthcare), allowing participants to navigate, measure, and analyze the studies as in routine practice. Each case was accompanied by basic clinical information (age, sex, chief complaint, height, and weight), and the TTE images retained the measurements originally performed by the sonographer. Participants were permitted to perform additional measurements using the PACS tools as needed. No standardized reporting template was imposed; participants generated reports freely, following the reporting style of their respective training institutions. The reference standard for each case was established by consensus reading by two board-certified cardiologists with > 10 years of echocardiography experience (YE Yoon and GY Cho). Specifically, step 1 reference categories and grading thresholds were assigned according to the current clinical guidelines specified in **Table S3**.

### Analytic approach

Continuous variables are presented as median (range), and categorical variables as count (percentage). Given the limited sample size per training level (level 1, n = 3; level 2, n = 5; and level 3, n = 3), nonparametric methods were used throughout. Baseline characteristics were compared across training levels by Fisher exact test (categorical) or the Kruskal–Wallis test (continuous and ordinal). Trends in safety, precision, quality, and composite scores across the three training levels (level 1 < level 2 < level 3) were tested using the one-sided Jonckheere-Terpstra trend test. Convergent validity of the framework was examined by Spearman rank correlation between precision and quality scores at both the case level and the participant level. Step 0 fail cases were assigned a precision score of 0 and a quality score of 0 in all level-based comparisons; domain-level precision scores were calculated using step 0 pass cases only, to characterize per-item performance independent of the global step 0 penalty. Per-item major-error rates and the distribution of quality grades were summarized descriptively. A two-sided P < 0.05 was considered statistically significant; trend test P-values are one-sided. All analyses were performed using R ver. 4.3 (R Foundation for Statistical Computing).

### EchoPeer performance across training levels

**Fig. 2:**
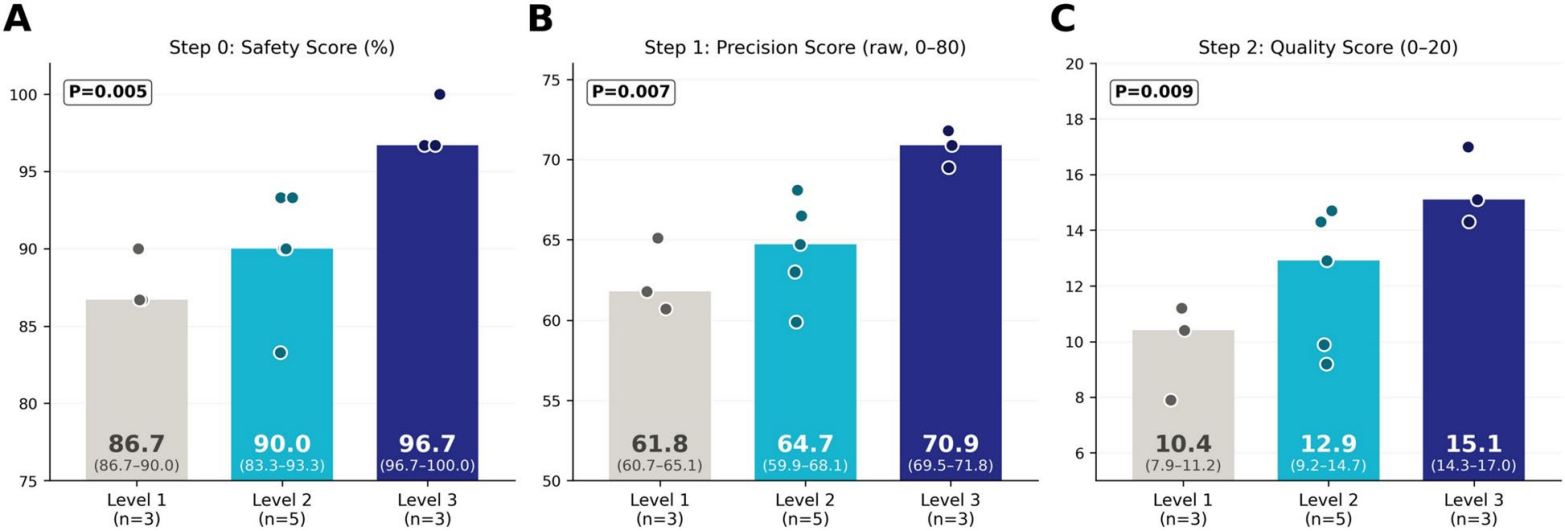
EchoPeer scores by training level. Bars represent median values; dots are individual participants. Numbers inside each bar give the median (range). **A** Step 0 safety score (%).**B** Step 1 precision score (0–80 scale). **C** Step 2 quality score (0–20 scale). P-values are from the one-sided Jonckheere-Terpstra trend test

Among the 30 cases, 9 contained reference-defined critical findings. The safety score demonstrated a significant positive trend across training levels (P = 0.005, Jonckheere-Terpstra test) (Fig. 2A). Median safety scores were 86.7% (range, 86.7%–90.0%) for level 1, 90.0% (range, 83.3%–93.3%) for level 2, and 96.7% (range, 96.7%–100%) for level 3. Per-participant sensitivity for critical finding detection also increased significantly with training level (level 1, 55.6% [range, 55.6%–66.7%]; level 2, 66.7% [range, 44.4%–88.9%]; level 3, 88.9% [range, 88.9%–100%]; P = 0.007, Jonckheere-Terpstra test). When critical finding reads were pooled across participants within each training level, the overall per-case sensitivity for critical finding detection rose from 59.3% (16 of 27) in level 1 to 92.6% (25 of 27) in level 3. Failure rates by critical finding category and training level are provided in Table S4.

For step 1 precision assessment (0–80 scale, with step 0 fail cases scored 0), the precision score showed a significant positive trend across training levels (P = 0.007, Jonckheere-Terpstra test) (Fig. 2B). Median precision scores were 61.8 (range, 60.7–65.1) for level 1, 64.7 (range, 59.9–68.1) for level 2, and 70.9 (range, 69.5–71.8) for level 3. Domain-level analysis revealed that domain A (left heart structure and function) had the lowest accuracy (median, 82.2%); whereas domain D (aorta, pericardium, intracardiac mass, congenital heart disease) had the highest (94.8%); domain C (right heart and pulmonary artery) and domain B (valves) were intermediate (85.5% and 94.8%, respectively). Major errors were concentrated in three items: regional wall motion abnormality severity (31.4%), LV mass and geometry (23.9%), and LV diastolic function (12.3%).

**Fig. 3:**
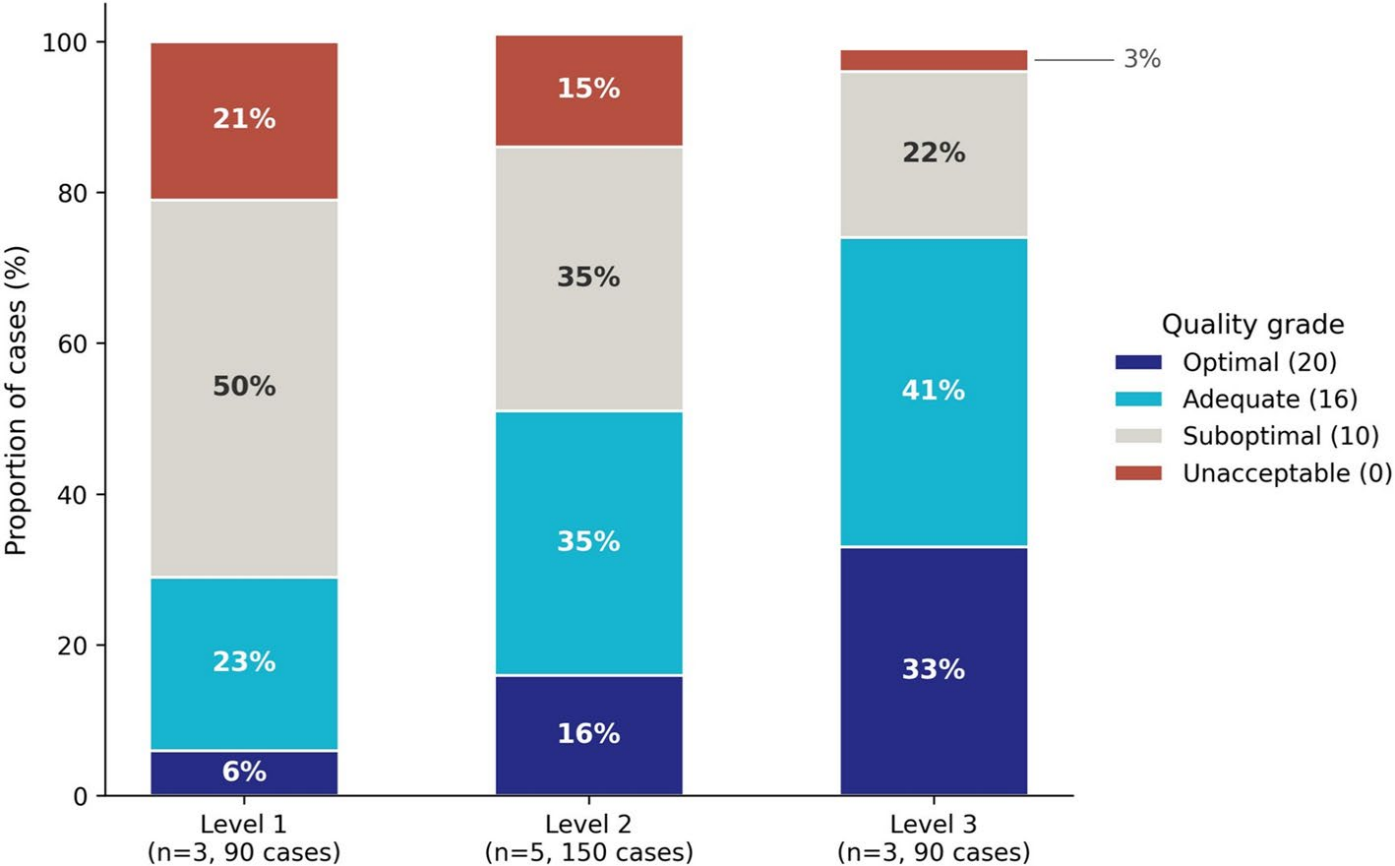
Step 2 quality grade distribution by training level. Stacked bars show the proportion of cases per quality grade within each training level (level 1, n = 90 cases; level 2, n = 150; level 3, n = 90). Step 0 fail cases were scored as “unacceptable.”

For step 2 quality assessment (0–20 scale), step 0 fail cases were assigned a quality score of 0 (unacceptable), consistent with the principle applied to step 1. The quality score also showed a significant positive trend across training levels (P = 0.009, Jonckheere-Terpstra test) (Fig. 2C). Median quality scores were 10.4 (range, 7.9–11.2) for level 1, 12.9 (range, 9.2–14.7) for level 2, and 15.1 (range, 14.3–17.0) for level 3. The distribution of quality grades showed a clear gradient across training levels (Fig. 3): the proportion of optimal grades increased from 6% in level 1 to 16% in level 2 and 33% in level 3, whereas the proportion of unacceptable grades decreased from 21 to 15% in level 2 and to 3% in level 3.

**Fig. 4:**
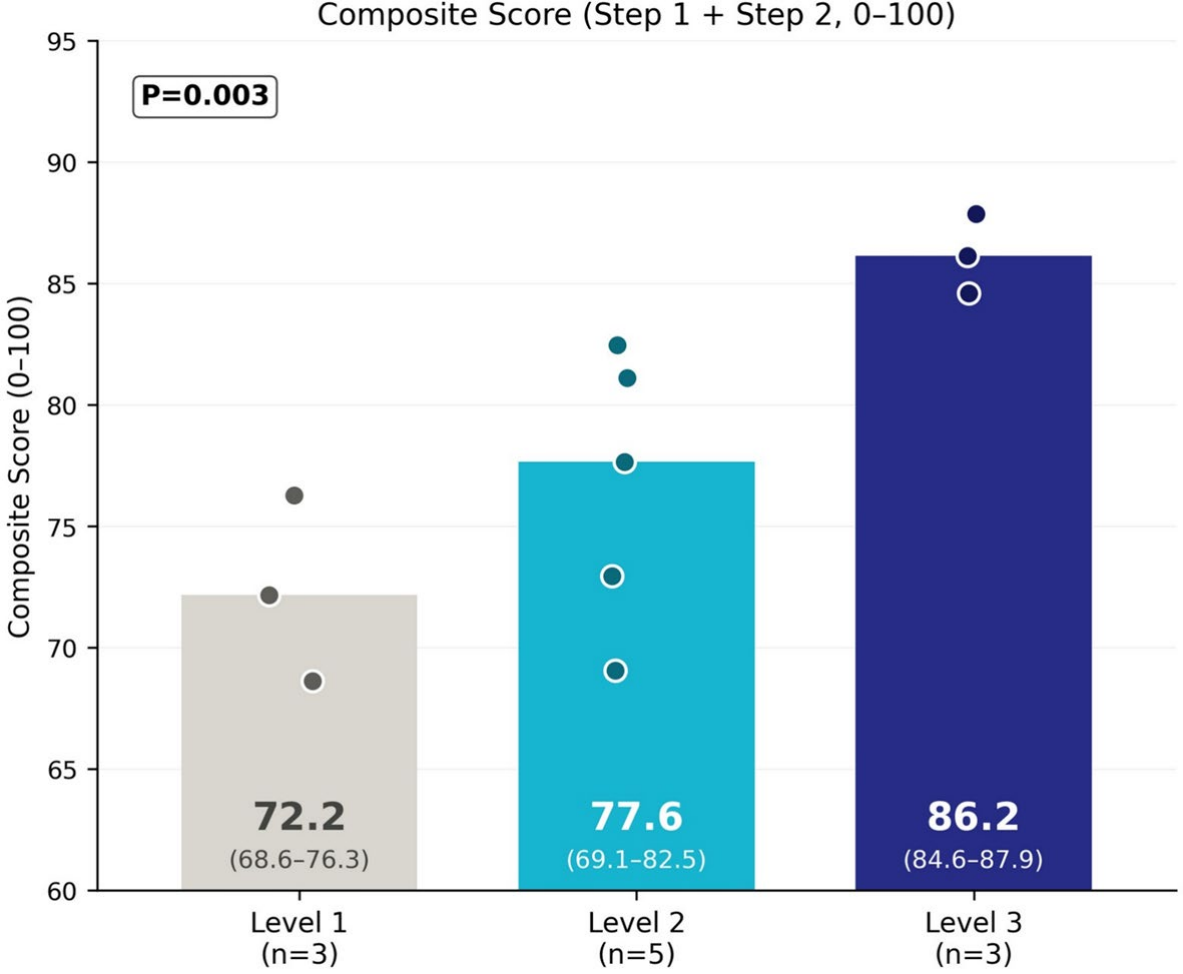
EchoPeer composite score by training level. Bars represent the median composite score; dots are individual participants. Numbers inside each bar give the median (range). The P-value is from the one-sided Jonckheere-Terpstra trend test

The EchoPeer composite score (0–100 scale), summing the precision and quality scores, also showed a significant positive trend across training levels (P = 0.003, Jonckheere-Terpstra test) (Fig. 4). Median composite scores were 72.2 (range, 68.6–76.3) for level 1, 77.7 (range, 69.1–82.5) for level 2, and 86.2 (range, 84.6–87.9) for level 3. Convergent validity of the EchoPeer framework was supported by significant positive correlations between precision and quality scores at both the case level (Spearman ρ = 0.568, P < 0.001) and the participant level (ρ = 0.888, P < 0.001), indicating that the two scoring components, while assessing different aspect of report performance, capture related dimensions of overall report quality.

## Discussion

EchoPeer was developed as a standardized framework for the evaluation of echocardiography reports, whether generated by an AI system or written by a human reader, against a consensus reference standard. The need for such a framework has increased as AI systems have moved from isolated measurement tasks toward full-report generation [22–24]. Existing evaluation frameworks address related but distinct purposes (Table 1). The RADPEER program of the American College of Radiology provides a discrepancy grading system for inter-reader peer review of radiology reports but was not designed for AI evaluation [11]. COCATS 4 defines training tiers for human cardiology fellows and is inapplicable to algorithms [14]. The 2025 ASE reporting standards specify the expected content of an adult TTE report but do not provide a quantitative scoring scheme [15]. To address this gap, the AI and Future Strategy Committee of the Korean Society of Echocardiography developed EchoPeer, a protocol designed to quantitatively evaluate adult TTE reports, whether AI-generated or human-authored, against a reference standard. EchoPeer is complementary to existing frameworks, evaluating reports in three sequential steps to produce a unified, quantitative profile of report performance: a safety gate (step 0), weighted item-level diagnostic precision (step 1), and report-level clinical utility (step 2). Each step produces an independent score and addresses a distinct clinical question about whether a report is safe, accurate, and usable.

The present study reports not only the development of this protocol but also its first pilot validation, conducted in cardiology fellows and junior staff members across three training levels. All three scores (safety, precision, and quality) increased monotonically with training level, and the observed error pattern reproduced difficulties already well characterized in echocardiographic interpretation. To our knowledge, EchoPeer is among the first frameworks to evaluate the safety, diagnostic precision, and clinical utility of echocardiography reports against a reference standard using a unified scoring approach. The following sections discuss the rationale for each protocol step, considering the pilot findings and their implications for the evaluation of AI-generated reports.

### Step 0 and the clinical imperative of a safety gate

The most consequential failure of an echocardiography report is not an imprecise measurement but the omission or fabrication of a finding that requires immediate action. This category of error is qualitatively different from a graded inaccuracy. An evaluation that begins by averaging item-level accuracy cannot represent it, because a report that is precise on every routine item but wrong on one critical finding would still receive a high score. Diagnostic error of this kind remains a substantial source of preventable harm, and in imaging it is interpretive rather than technical error that predominates [25, 26]. The conditions screened at step 0, including cardiac tamponade, AAS, the mechanical complications of MI, acute PE, and severe valve obstruction, share the property that a single missed or invented diagnosis can determine whether a patient receives timely and appropriate treatment [27–29]. EchoPeer therefore places a safety gate ahead of any accuracy scoring, so that a critical omission or a critical hallucination is identified before, and independently of, the diagnostic detail of the report. The step 0 critical finding list is not intended to be exhaustive; additional conditions, such as severe LV systolic dysfunction in the setting of cardiogenic shock, may be incorporated in future EchoPeer versions or institution-specific adaptations.

The pilot validation illustrated the practical importance of this gate. Although critical finding sensitivity improved with training, residual failures concentrated in a small number of diagnoses recognized as clinically challenging, most notably low-flow, low-gradient severe aortic stenosis and acute PE. In this human cohort, all step 0 failures were critical omission; no critical hallucinations occurred at any training level. However, this pattern does not necessarily predict the behavior of AI systems, since generative models may reproduce routine-reporting patterns while failing in uncommon but high-risk scenarios [30–33]. For example, LLMs can introduce unsupported or fabricated statements when summarizing clinical text [34–36]. EchoPeer was designed from the outset to detect both failure modes: step 0 screens each report for critical omission and critical hallucination simultaneously. This dual screening is expected to be especially relevant for AI-generated reports, in which a fluently written report may nonetheless be critically incomplete or fabricated; step 0 identifies such reports as unsafe before any accuracy-based evaluation is applied.

### Step 1 and item-level diagnostic precision

A report that passes the safety gate is not thereby clinically adequate, because its value to the referring physician depends on the accuracy of each finding that informs management. The severity of valvular disease, the presence and extent of a regional wall motion abnormality, and the grade of diastolic dysfunction each carry direct therapeutic consequences, and an evaluation that records only an overall impression cannot show where a report is reliable and where it is not. Even among trained readers, the reproducibility of echocardiographic interpretation is limited, particularly for visual assessment of regional wall motion and for diastolic function grading [37–39]. Step 1 was therefore designed to resolve diagnostic accuracy at the level of the individual item, weighting each item by its clinical importance and grading it against the reference standard so that the resulting score reflects clinically meaningful agreement rather than verbatim concordance.

In the pilot validation, major errors clustered in the left heart domain, particularly in regional wall motion abnormality severity, LV mass and geometry, and diastolic function. These items share a common feature: they depend either on subjective visual analysis or on the integration of multiple parameters according to guideline-recommended algorithms, both of which are recognized weaknesses of human reporting [40–42]. Interestingly, the challenges that AI reporting systems face in these areas are not identical. Items requiring visual analysis have been the focus of dedicated AI tools, such as those developed for regional wall motion [17, 43, 44], and for the recognition of specific phenotypes such as cardiac amyloidosis [45, 46]. These systems are likely to encounter difficulties analogous to those of human readers, and the translational implication is that AI validation effort should be directed toward such items, with the most demanding acceptance thresholds. In contrast, items requiring multiparameter integration according to fixed algorithms, such as LV mass and geometry, or diastolic function [18, 19], are inherently well suited to systematic algorithm execution [47–49], and the same applies to severity grading in valvular heart disease, which relies on established numeric cutoffs [17, 50]. Competent performance on such items, however, does not by itself establish that an AI reporting system is ready for independent clinical use, and structured validation through a framework such as EchoPeer remains necessary across all item categories.

### Step 2 and the clinical utility of the report

Diagnostic accuracy, even when complete, does not by itself make a report clinically useful. A report serves the referring physician only if it answers the clinical question that prompted the study, integrates its findings without internal contradiction, and states its conclusion clearly enough to be acted upon. Structured reporting has been advocated across imaging specialties precisely because unstructured narrative is an inconsistent vehicle for these properties [20, 51]. In echocardiography, the quality of the report is recognized as an important component of laboratory quality, distinct from the accuracy of any single measurement [21]. Step 2 was designed to capture this dimension through three checkpoints (relevance, synthesis, and clarity) that correspond to the criteria by which a referring physician judges whether a report can be acted upon without further clarification.

In the pilot validation, the distribution of quality grades shifted consistently with training, with the proportion of reports judged optimal increasing and the proportion judged unacceptable falling across successive levels. This pattern supports the value of evaluating precision and quality as separate dimensions: a report may be diagnostically accurate at the item level but still fail to provide a coherent, actionable interpretation. The distinction is particularly relevant for AI-generated reports, as LLMs produce text that is fluent and persuasive irrespective of its correctness [52, 53]; fluent language can conceal both fabrication and disorganization, and early applications of generative models to echocardiographic reporting illustrate both the promise and this hazard [54].

### An integrated framework for AI-generated and human-authored reports

The three steps were designed to operate not as independent audits but as a single instrument, and the pilot validation supports that design. Each of the three scores (safety, precision, and quality) increased monotonically with training level. Because training level was an external marker of competence established independently of the protocol, this monotonic increase provides preliminary evidence for the framework's construct validity. The integrated design also requires that step 0 failures be retained, rather than being removed. Safety failures and item-level inaccuracy were not independent properties of a report and were both more frequent among the least experienced participants; excluding step 0 fail cases from step 1 and step 2 analyses would therefore disproportionately remove the most demanding cases from those participants and obscure the gradient the protocol is intended to measure. Assigning a precision score of 0 and a quality score of 0 to step 0 fail cases preserves the penalty for critical finding failure within the integrated score and prevents bias in ranking competence.

By grading safety, diagnostic precision, and clinical utility on a common scale, EchoPeer allows a report to be evaluated in the same terms regardless of its author, and this report-centered design is what extends the framework to AI-generated reports. The need for such an instrument is increasingly clear. Reporting and evaluation standards for medical AI have advanced quickly, from trial-level guidance [55] to frameworks for the early-stage clinical evaluation of decision-support systems [56], the reporting of prediction models [57], and risk-proportionate regulatory assessment [58]. These frameworks govern how an AI system is studied and reported; EchoPeer is complementary, specifying what an acceptable echocardiography report must contain and how far a given report falls short. For AI-generated echocardiography reports, step 0 may be especially important because a fluent text can still omit a life-threatening finding or introduce a fabricated diagnosis. Step 1 can then localize the source of error to specific echocardiographic domains and items, distinguishing failures of visual interpretation from failures of guideline-based integration, while step 2 assesses whether the generated report answers the clinical question, integrates findings coherently, and communicates uncertainty appropriately. A standard of this kind, validated first against a graded range of human competence, provides the reference against which the safety and adequacy of an automated report can be judged as such systems move toward clinical deployment.

### Limitations

Several limitations of this study warrant consideration. First, this was a small pilot study with a limited number of participants, and the number within each training level was small; the trends reported here therefore require confirmation in a larger multicenter cohort. Second, the protocol was validated in human readers rather than in AI systems, and its performance on AI-generated reports, the ultimate intended application, remains to be established; the human cohort was studied deliberately to provide a graded benchmark of competence before the framework is applied to AI. Third, the reference standard was established by consensus of two experienced cardiologists, which may not capture the full uncertainty inherent in the reference standard definition; future studies could employ a larger expert panel or external core laboratory adjudication. Fourth, EchoPeer relied on limited expert input at two stages: the step 1 item weights were an initial expert-derived operational scale rather than derived from externally validated multiexpert consensus, and each report was scored by a single expert cardiologist without formal interrater reliability assessment; future studies should broaden expert calibration and quantify interrater agreement. Fifth, the case set was enriched for step 0 critical findings and for a range of pathologies, which strengthens content coverage but does not reproduce the disease prevalence of routine practice, so the absolute scores should not be interpreted as estimates of real-world performance. Sixth, the study was conducted at Korean training hospitals, and reporting conventions, terminology, and clinical communication norms may differ in other healthcare systems; the framework anticipates this by allowing institution-specific adaptation of grading thresholds in step 2, but international validation will be needed before broader application. Finally, the current framework version focuses on adult TTE; extensions to TEE, stress echocardiography, 3D imaging, and strain reporting will require dedicated validation in future work. Future validation studies should also include human- and AI-generated reports spanning a deliberately defined spectrum of agreement with the reference standard, from near-concordant reports to reports with clinically meaningful discrepancies or major errors, to further calibrate the scoring framework and test its discrimination across levels of report accuracy.

## Conclusions

This study reports the development of EchoPeer, a three-step protocol that grades the safety, diagnostic precision, and clinical utility of AI-generated and human-authored echocardiography reports, together with a first pilot validation in cardiology fellows and junior staff members across three training levels. All three scores increased monotonically with training level, and the error pattern reproduced difficulties already characterized in echocardiographic interpretation, supporting the validity of EchoPeer as a measurement instrument. EchoPeer offers a practical, clinically grounded standard for evaluating echocardiography reports regardless of their author. Larger multicenter studies, including the direct assessment of AI-generated reports, will be needed to confirm its applicability and refine its role in the broader landscape of AI reporting validation.

## Supplementary Information

Additional file 1: Table S1. Detailed step 0 safety assessment: critical findings, clinical definitions, and fail criteria. Table S2. Step 1 precision assessment: scoring scale and operational rules. Table S3. Complete step 1 precision assessment: 25 items, weights, reference categories, and item-level scoring principles. Table S4. Step 0 failure rates by critical finding category and training level. Appendix S1. Self-assessment questionnaire.

## Data Availability

No datasets were generated or analysed during the current study.

## Abbreviations

AAS: Acute aortic syndrome
AI: Artificial intelligence
ARVC: Arrhythmogenic right ventricular cardiomyopathy
ASE: American Society of Echocardiography
DCM: Dilated cardiomyopathy
HCM: Hypertrophic cardiomyopathy
LLM: Large language model
LV: Left ventricular
MI: Myocardial infarction
NA: Not applicable
NDLVC: Nondilated left ventricular cardiomyopathy
PACS: Picture archiving and communication system
PE: Pulmonary embolism
RCM: Restrictive cardiomyopathy
RV: Right ventricular
TEE: Transesophageal echocardiography
TTE: Transthoracic echocardiography
