# Supplementary material for: ﻿Special article—EchoPeer: a standardized framework for assessing echocardiography reports in the era of artificial intelligence: a recommendation from the Korean Society of Echocardiography AI and Future Strategy Committee
Source: J Cardiovasc Imaging. 2026 Aug 14;34:23. doi: 10.1186/s44348-026-00085-6 (PMC13474789; doi:10.1186/s44348-026-00085-6)
Supplement: Supplementary file 1 [file 44348_2026_85_MOESM1_ESM.docx]

**Supplementary Materials**

***Special Article — EchoPeer: A Standardized Framework for Assessing Echocardiography Reports in the Era of Artificial Intelligence: A Recommendation from the Korean Society of Echocardiography AI and Future Strategy Committee***

**Table of contents**

**Supplementary Table S1.** Detailed Step 0 Safety Assessment: Critical Findings, Clinical Definitions, and Fail Criteria

**Supplementary Table S2.** Step 1 Precision Assessment: Scoring Scale and Operational Rules

**Supplementary Table S3.** Complete Step 1 Precision Assessment: 25 Items, Weights, Reference Categories, and Item-Level Scoring Principles

**Supplementary Table S4.** Step 0 Failure Rates by Critical-Finding Category and Training Level

**Supplementary Appendix S1.** Self-Assessment Questionnaire

**References**

**Supplementary Table S1. Detailed Step 0 Safety Assessment: Critical Findings, Clinical Definitions, and Fail Criteria**

| **No.** | **Critical Finding** | **Clinical Definition** | **Omission Fail Criteria  (GT-positive but missed)** | **Hallucination Fail Criteria (GT-negative but asserted)** |
| --- | --- | --- | --- | --- |
| 1 | **Suspected cardiac tamponade** | Tamponade physiology with right atrial or right ventricular collapse and inferior vena cava plethora. Simple pericardial effusion without hemodynamic compromise is not included. | Reference-positive but unmentioned, or asserted as “no tamponade / no effusion”. | Reference-negative but asserted as “tamponade present” or “probable/suspected” without an explicit uncertainty marker. |
| 2 | **Suspected aortic dissection or acute aortic syndrome** | Aortic dissection, intramural hematoma, or penetrating atherosclerotic ulcer suggested or confirmed.. | Reference-positive but unmentioned, or asserted as “aorta normal / no dissection”. | Reference-negative but asserted as “acute aortic syndrome/dissection present” or “probable/suspected” without an explicit uncertainty marker. |
| 3 | **Complications of myocardial infarction** | Ventricular septal rupture, free-wall rupture, or papillary muscle rupture, or pseudoaneurysm requiring immediate intervention or close clinical attention. | Reference-positive but unmentioned, or asserted as “no complication”. | Reference-negative but asserted as “rupture or pseudoaneurysm present” or “probable/suspected” without an explicit uncertainty marker. |
| 4 | **Thrombus in transit** | Mobile thrombus in the right-sided chambers (RA, RV, IVC, SVC) with high risk of acute pulmonary embolism. Left-sided thrombi (LA, LV) are evaluated under the Step 1 intracardiac mass and thrombus item rather than as a Step 0 critical finding. | Reference-positive but unmentioned, or asserted as “no thrombus”. | Reference-negative but asserted as “thrombus in transit present” or as “probable/suspected” without an explicit uncertainty marker. |
| 5 | **Acute RV dysfunction and suspected acute pulmonary embolism** | Acute RV pressure overload (RV dilation with dysfunction, D-shaped interventricular septum) suggesting acute pulmonary embolism. | Reference-positive (acute pulmonary embolism confirmed by cross-modality imaging or clinical context, or equivalent acute RV dysfunction), but the report asserts “right ventricle normal”, or contains no mention of acute right ventricular strain. | Reference-negative but asserted as “pulmonary embolism present” or as “probable/suspected” without an explicit uncertainty marker. |
| 6 | **LV assist device or veno-arterial extracorporeal membrane oxygenation complication** | Acute LVAD or VA-ECMO complication requiring immediate management (cannula malposition, suction events, thrombus, or flow abnormalities). | Reference-positive but unmentioned, or asserted as “no device issue”. | Reference-negative but asserted as “device complication present” or “probable/suspected” without an explicit uncertainty marker. |
| 7 | **Severe valve obstruction/stenosis in prosthetic or native valves** | Severe valve stenosis or obstruction of any cause (calcific, thrombotic, vegetation, mass, or prosthetic dysfunction). Acute or new presentations carry the highest priority for missed diagnosis. | Reference-positive but unmentioned, or asserted as non-severe / normal. | Reference-negative but asserted as “severe obstruction present” or “probable/suspected” without an explicit uncertainty marker. |

Critical findings were adopted from Table 14 of the 2025 ASE Guidelines for the Standardization of Adult Echocardiography Reporting¹⁴⁾ and may be revised in future framework versions as evidence and guidelines evolve. The Step 0 architecture — Pass/Fail screening with critical omission and critical hallucination as the two failure modes — remains stable across versions.

Reports that explicitly mark uncertainty (e.g., "cannot exclude X, suggest further evaluation") are not classified as failures, even when "probable" or "suspected" language is used together with such markers.

When TTE findings are equivocal or technically limited, the reference may be established by confirmatory cross-modality imaging (cardiac CT, CMR, or TEE), invasive testing, or surgical findings.

A case is classified as Fail at Step 0 if any of the seven findings fail for that case; otherwise, the case passes Step 0.

Abbreviations: IVC, inferior vena cava; LA, left atrium; LV, left ventricle; LVAD, left ventricular assist device; RA, right atrium; RV, right ventricle; SVC, superior vena cava; VA-ECMO, veno-arterial extracorporeal membrane oxygenation.

**Supplementary Table S2. Step 1 Precision Assessment: Scoring Scale and Operational Rules**

Rules below apply hierarchically: Step 0 propagation overrides Step 1 scoring; N/A removes the item from scoring before any other rule; Indeterminate is evaluated before Implicit Normal; Implicit Normal is applied only when the reference is not Indeterminate and no internal inconsistency exists.

| **Component** | **Definition or Rule** |
| --- | --- |
| **Scoring scale (5 tiers)** | Each item is graded on one of five tiers and scored as a fraction of its weight (W):   - **Exact** (W × 1.00) - report matches the reference grade or category exactly. - **Acceptable** (W × 0.75) – *default rule*: one adjacent grade difference on ordered ordinal scales, without altering clinical management. Item-specific exceptions are detailed in Supplementary Table S3. - **Discrepancy** (W × 0.50) - *default rule:* non-adjacent (≥2 grade) mismatch, two grades, at a level that may change management; or omission of clinically relevant abnormal finding (item-specific threshold) - **Major Error** (W × 0.25) - direction-reversal error (e.g., severe ↔ none); active denial of reference-positive major finding; false-positive assertion of a major finding; or omission of a critical reference-positive finding (item-specific) - **Not Applicable** (N/A)- reference is missing or item is excluded by protocol; remove from the denominator |
| **Adjacent-grade rule** | On ordered scales (e.g., none/mild/moderate/severe), a one-step difference is normally graded as Acceptable. However, item-specific adjacent rules in Supplementary Table S3 may modify this default, particularly for items where the clinical implication of a grade change is high |
| **Implicit Normal handling**  **(no mention = normal)** | Applied only when *all three* conditions hold: (1) reference is normal or absent, (2) report contains no contradicting or ambiguous cue for that item, and (3) no internal contradiction exists within the report. Not applied when the reference is Indeterminate.  **Item-specific mapping:** • **Implicit = Exact** — LV cavity size; valvular AS / AR / MS / MR / TR (reference none or trace); RV size and function; aorta; intracardiac mass and thrombus; simple and complex congenital heart disease; prosthetic valve presence • **Implicit = Acceptable** — RWMA presence and distribution; LA size; pulmonary hypertension (reference normal or low probability); IVC and RA pressure; pericardial effusion (reference none or trivial) • **Implicit prohibited (no mention = Omission)** — global LVEF; RWMA severity (when triggered); LV diastolic function (in eligible cases). For these items, omission triggers Discrepancy or Major Error based on the reference severity, per item-specific rules in **Supplementary Table S3**. |
| **Indeterminate handling** | Triggered on the report side by limiting or evasive phrases (“cannot assess/limited/not well visualized/cannot exclude”):   - Reference Indeterminate + report Indeterminate → Exact - Reference Indeterminate + report unmentioned → Discrepancy - Reference not Indeterminate + report provides an evidence-based technical caveat → Acceptable - Reference not Indeterminate + report uses a vague, evasive caveat → Discrepancy   (Or Major Error if it actively masks a reference-positive major finding in Step 1) |
| **Internal inconsistency** | When a single report contains contradictory statements for the same item (e.g., body text states “no effusion” but the conclusion states “moderate effusion”), the worst-direction statement is used for Step 1 grading. The clinical risk and synthesis quality of the inconsistency itself are evaluated separately under Step 2 Synthesis. |
| **N/A categories** | Two types, both removed from the Step 1 dominator:   - N/A (No-data) – reference standard is completely unobtainable due to imaging dropouts or protocol limitations. “Available but limited image quality” is handled under Indeterminate, not N/A. - N/A (Excluded) - protocol-driven exclusions: native-valve items in the presence of a prosthetic valve; surgically altered cardiac anatomy; untriggered conditional items; non-target scope |
| **Cardiomyopathy phenotype trigger** | When the reference establishes a cardiomyopathy phenotype (HCM, DCM, NDLVC, ARVC, or RCM), the LV mass and geometry item is restricted to assessing the objective presence of LVH and LV dilation only. Phenotype framing itself is evaluated at Step 2 (Relevance and Synthesis). |

Abbreviations: AR, aortic regurgitation; ARVC, arrhythmogenic right ventricular cardiomyopathy; AS, aortic stenosis; DCM, dilated cardiomyopathy; HCM, hypertrophic cardiomyopathy; IVC, inferior vena cava; LA, left atrium; LV, left ventricle; LVEF, left ventricular ejection fraction; LVH, left ventricular hypertrophy; MR, mitral regurgitation; MS, mitral stenosis; N/A, not applicable; NDLVC, non-dilated left ventricular cardiomyopathy; PH, pulmonary hypertension; RA, right atrium; RCM, restrictive cardiomyopathy; RV, right ventricle; RWMA, regional wall motion abnormality; TR, tricuspid regurgitation; W, item weight.

**Supplementary Table S3. Complete Step 1 Precision Assessment: 25 Items, Weights, Reference Categories, and Item-Level Scoring Principles**

Reference categories and grading thresholds in this table reflect the current framework version, anchored to the clinical guidelines listed in the Methods. They may be revised in future versions as evidence and guidelines evolve. Operational scoring rules (5-tier scale, Adjacent-grade rule, Implicit Normal handling, Indeterminate handling, Internal inconsistency, N/A categories, Cardiomyopathy phenotype trigger) are defined in **Supplementary Table S2**.

| **Item** | | **W** | **Ground-Truth Categories** | **Core Scoring Principle** |
| --- | --- | --- | --- | --- |
| **Domain A. Left Heart Structure and Function (22 points)** | | | | |
| **A1** | **LV cavity size** | 4 | Reduced / Normal / Mildly enlarged / Moderately enlarged / Severely enlarged^1)^ | - **Implicit normal:** reference normal, unmentioned → Acceptable - **Omission:** reference reduced or any enlarged grade, unmentioned → Discrepancy - **Direction reversal:** reference reduced ↔ report enlarged; reference enlarged ↔ report reduced → Major Error |
| **A2** | **LV mass and geometry** | 2 | 8-pattern classification (normal/physiologic hypertrophy/concentric remodeling/eccentric remodeling/concentric hypertrophy/mixed hypertrophy/dilated hypertrophy/eccentric hypertrophy) based on LVVi, LVMi, and RWT^2)^ | **CMP Trigger ON** (reference Disease Category =HCM, DCM, NDLVC, ARVC, RCM) ^3)^: only LVH presence and LV dilation are scored   - HCM cases: report HCM (any subtype) → Exact; other CMP named → Major Error; CMP absent → Discrepancy - Other CMP: matching CMP named → Exact; different CMP confirmed → Major Error; CMP unmentioned → axis scoring applied   **CMP Trigger OFF (Non-CMP)**   - **Implicit normal**: reference normal + geometry unmentioned → Acceptable - **Omission:** reference ≠ normal, geometry completely unmentioned → Discrepancy - **Axis reversal** (LVH or LV dilation) → Major Error |
| **A3** | **Global LV systolic function (LVEF)** | 4 | Hyperdynamic / Normal / Mildly reduced / Moderately reduced / Severely reduced (sex-specific cutoffs)^1)^ | - **Omission:** reference normal or hyperdynamic, unmentioned → Discrepancy; reference mildly reduced or worse, unmentioned → Major Error - **Direction reversal:** reference severely reduced ↔ report Normal/Hyperdynamic → Major Error |
| **A4** | **RWMA presence and distribution** | 4 | (A) Status (none/present/indeterminate)  (B) Pattern type (coronary-territory / non-coronary / mixed)  (C) Distribution (LAD/LCX/RCA ± apex; or non-coronary descriptor: apical ballooning, mild ventricular, basal, diffuse/global, patchy/multifucal) | - **Implicit none:** reference none, unmentioned with no contradicting cue → Acceptable - **Omission**: reference present, unmentioned → Discrepancy - **Active denial/false-positive major finding:** reference present with extensive coronary or non-coronary focal pattern, asserted as "no RWMA; reference none, asserted as "extensive/multivessel RWMA” → Major Error |
| **A5** | **RWMA severity** | 2 | Mild (regional hypokinesia) / Severe (regional akinesia or dyskinesia). Triggered only when A4 = present or indeterminate | - **Not triggered** (excluded from denominator): A4 = none, or global hypokinesia only - **Omission:** reference mild, unmentioned → Discrepancy; reference severe, unmentioned → Major Error - **Active denial/false-positive major finding:** reference severe, asserted as "no RWMA"; reference none, asserted as "definite dyskinesia / aneurysmal motion" → Major Error |
| **A6** | **LA size** | 2 | Normal / Mildly enlarged / Moderately enlarged / Severely enlarged^1)^ | - **Implicit normal**: reference normal or mildly enlarged, unmentioned → Acceptable - **Omission**: reference moderately or severely enlarged, unmentioned → Discrepancy - **Direction reversal:** reference severely enlarged ↔ report "normal LA size"; reference normal ↔ report "severely/markedly enlarged" → Major Error |
| **A7** | **LV diastolic function** | 4 | Two tracks: **Eligible** (Normal/Grade 1/Grade 2/Grade 3/Indeterminate) and **Excluded condition** (atrial fibrillation, significant MR, any MS, moderate-to-severe MAC, heart transplantation, LVAD, noncardiac PH, pericardial constriction)^4)^ | **Eligible track**   - **Omission:** reference normal → Acceptable; Grade 1 or Indeterminate → Discrepancy; Grade 2–3 → Major Error - **Direction reversal:** reference normal ↔ report "restrictive/Grade 3"; reference Grade 3 ↔ report "normal" → Major Error   **Excluded track**   - **Appropriate handling:** omission, or applicability state with reason → Exact - **Formal grading imposed despite excluded condition:** Grade 1 or 2 → Discrepancy; Grade 0 or 3 → Major Error |
| **Domain B. Valves (28 points)** | | | | |
| **B1** | **Aortic valve morphology** | 2 | Tricuspid (TAV)/Bicuspid (BAV)/Other native (unicuspid or quadricuspid)/Indeterminate. Prosthetic AV(SAVR/TAVR) → N/A (Excluded). | - **Implicit TAV**: reference TAV, unmentioned with no BAV/Other cue and no morphology-limitation phrase → Exact - **Omission**: reference BAV or other, unmentioned without strong cues (raphe/two cusps/fusion/Sievers) → Discrepancy - **Direction reversal**: reference TAV ↔ report “definite BAV”; reference BAV ↔ report "definite tricuspid valve" or "normal aortic valve morphology" → Major Error |
| **B2** | **Aortic stenosis severity** | 4 | No AS/Mild/Moderate/Severe/Very severe (with intermediate range allowed)/Suspicious LFLG severe (with subtypes: classical/paradoxical/normal-flow low-gradient). Prosthetic AV → N/A (Excluded)^5, 6)^ | - **Implicit normal**: reference normal/no AS, unmentioned → Exact - **Omission**: reference mild or worse, unmentioned → Discrepancy - **Direction reversal**: reference severe or very severe ↔ report "no AS/mild AS" definitive; reference Normal or Mild ↔ report "severe/critical AS" definitive; reference Suspicious LFLG severe ↔ report "no AS / mild AS" definitive → Major Error   **Suspicious LFLG severe handling** (preserves the "severe possibility requiring further evaluation")   - **Subtype-matched suspicion**: reference suspicious LFLG severe ↔ report "LFLG severe AS suspected/possible" with matching subtype → Exact - **Unspecified subtype**: reference suspicious LFLG severe ↔ report "LFLG severe AS suspected/possible" without subtype, or range "moderate-to-severe / at least moderate" → Acceptable - **Subtype mismatch alone** (correct LFLG-severe suspicion, wrong subtype) → not penalized - **Fixed as not-severe**: reference suspicious LFLG severe, report asserts mild or moderate definitively → Discrepancy - **Active denial**: reference Suspicious LFLG severe, asserted as "no AS / mild AS" definitive → Major Error |
| **B3** | **Aortic regurgitation severity** | 2 | None or trace/Mild/Moderate/Severe (intermediate ranges allowed). Prosthetic AV→ N/A (Excluded)^5)^ | - **Implicit normal:** reference none/trace, unmentioned → Exact - **Implicit mild:** reference mild, unmentioned → Acceptable - **Omission:** reference moderate or worse, unmentioned → Discrepancy - **Direction reversal**: reference severe (or moderate-to-severe) ↔ report "no AR/trace / trivial", or reference none/trace ↔ report "severe AR" → Major Error |
| **B4** | **Mitral valve morphology** | 2 | Normal/ Degenerative (calcific/MAC)/prolapse or flail/ Rheumatic/Functional/Congenital/Mixed /Indeterminate. Prosthetic MV, repair, or TEER → N/A (Excluded). | - **Implicit normal:** reference normal + etiology unmentioned + no false-positive etiology label → Exact - **Implicit none with low-severe MR:** reference single etiology with MR ≤ mild and no high-risk lesion/congenital anomaly/rheumatic features, etiology unmentioned → Exact - **Omission with trigger:** reference MR ≥ moderate, or high-risk lesion (flail/chordal rupture/perforation), or congenital anomaly, or rheumatic + MS, etiology unmentioned → Discrepancy - **Etiology mismatch** (within acquired etiologies) → Discrepancy - **False-positive etiology**: reference normal, report asserts acquired etiology label → Discrepancy - **Active denial**: reference flail/chordal rupture/perforation, asserted as functional MR or "no structural abnormality"; or reference confirmed congenital anomaly, asserted as "normal MV anatomy / no congenital anomaly" → Major Error - **False-positive major finding**: reference native, report asserts definitive prosthesis/repair → Major Error |
| **B5** | **Mitral stenosis severity** | 4 | No MS/Mild/Moderate/ Severe. Prosthetic MV, repair, or TEER → N/A (Excluded)^7)^ | - **Implicit normal**: reference no MS, unmentioned → Exact - **Omission**: reference mild or worse, unmentioned → Discrepancy - **Wrong attribution**: reference MS, report describes MS indicators but attributes to MR or tachycardia → Discrepancy - **Direction reversal**: reference severe ↔ report "no MS / not stenotic", or reference no MS or trivial ↔ report "severe/critical MS" → Major Error |
| **B6** | **Mitral regurgitation severity** | 4 | None or trace/Mild/Moderate/Severe. Prosthetic MV or TEER, N/A. Prosthetic MV, repair, or TEER → N/A (Excluded).^5)^ | - **Implicit normal**: reference none/trace, unmentioned → Exact - **Implicit mild**: reference mild, unmentioned → Acceptable - **Omission**: reference moderate or worse, unmentioned → Discrepancy - **Direction reversal**: reference severe (or moderate-to-severe) ↔ report "no MR/trace", or reference none/trace ↔ report "severe MR" → Major Error |
| **B7** | **Tricuspid regurgitation severity (including Ebstein and congenital TV)** | 4 | (A) TR severity (TRVARC 6-tier): None or trace/Mild/Moderate/Severe/Massive/ Torrential. (B) Ebstein/congenital TVanomaly (trigger-based): none/present/indeterminate. Prosthetic TV or TV repair, or TEER → N/A (Excluded). Final item score = worst rating across scored axes.^8)^ | **TR severity (Axis A)**   - **Implicit normal**: reference none/trace, unmentioned → Exact - **Implicit mild**: reference mild, unmentioned → Acceptable - **Omission**: reference moderate or worse, unmentioned → Discrepancy - **Direction reversal**: reference ≥severe (severe/massive/torrential) ↔ report "no TR/trace/trivial", or reference none/trace or mild ↔ report "torrential/massive TR" → Major Error   **Ebstein / congenital TV (Axis B; trigger-based)**   - **Triggered only** when reference present/indeterminate, or report explicitly mentions Ebstein/congenital TV anomaly, or indication includes Ebstein/CHD evaluation; otherwise N/A - **Conditional omission**: reference present, unmentioned, with TR ≥ moderate or report addresses TV morphology/CHD → Discrepancy - **Active denial**: reference present, asserted as "no Ebstein / normal TV insertion / no congenital TV anomaly" → Major Error - **False-positive major finding**: reference none, asserted as "definite Ebstein anomaly" → Major Error |
| **B8** | **Prosthetic valve presence, type, and location** | 2 | Per valve (AV/MV/TV/PV): Presence (yes/no); Location; Type (mechanical/bioprosthetic/repair device/repair device [annuloplasty ring, TEER, indeterminate). Scored per-valve; patient-level score = worst-of-valves. Dysfunction handled in B9. | - **Implicit none**: reference no prosthesis + prosthesis unmentioned → Exact - **Omission**: reference prosthesis present, report describes only native-valve context → Discrepancy - **Multiple-prosthesis under-spec**: reference ≥2 prostheses, report uses single phrase ("s/p valve replacement") without specifying each → Discrepancy - **Location or type mismatch** → Discrepancy - **Direction reversal (mechanical)**: reference no prosthesis ↔ report mechanical prosthesis confirmed; reference mechanical prosthesis ↔ report "no prosthetic/native valve" confirmed; or reference mechanical at one location ↔ report mechanical at a different location → Major Error |
| **B9** | **Prosthetic valve dysfunction** | 4 | Triggered only when the reference prosthesis is present. Dysfunction type (obstruction/stenosis/regurgitation [transvalvular or paravalvular leakage]/ dehiscence/endocarditis-related/mixed / none/indeterminate); and severity (none/ mild/moderate/severe; intermediate range allowed). Scored per-valve; patient-level score = worst-of-valves. | - **Type and severity match** → Exact - **Type match with adjacent severity** → Acceptable - **Type mismatch or wrong attribution**: reference prosthetic dysfunction described as native valve disease ("severe AS/severe MS") → Discrepancy - **Active denial**: reference moderate-to-severe or severe dysfunction (any type), asserted as "normal prosthesis / no dysfunction" → Major Error - **False-positive major finding**: reference none, asserted as "definite dehiscence", "severe obstruction", or "severe PVL" → Major Error |
| **Domain C. Right Heart and Pulmonary Artery (14 points)** | | | | |
| **C1** | **RV size and structure** | 2 | Composite of (A) RV size (normal / mildly/moderately/severely dilated) and (B) RV wall hypertrophy (RVH: normal/ mild/moderate/severe). Final score = weighted composite (RV size 0.7 + RVH 0.3).^9)^ | - **Implicit normal**: reference normal (RV size or RVH), unmentioned → Exact - **Implicit mild**: reference mildly dilated or mild RVH, unmentioned → Acceptable - **Omission**: reference moderate, unmentioned → Discrepancy; reference severe, unmentioned → Major Error - **Direction reversal**: reference severely dilated ↔ report "normal /no RV dilatation"; reference normal ↔ report "severe/marked RV dilatation"; same for RVH → Major Error |
| **C2** | **RV systolic function** | 4 | Normal/Mildly reduced/Moderately reduced/Severely reduced (TAPSE, S′, FAC, RVEF integrated; worst grade)^9)^ | - **Implicit normal**: reference normal, unmentioned with no RV dysfunction cue → Exact - **Implicit mild**: reference mildly reduced, unmentioned → Acceptable - **Omission**: reference moderately reduced, unmentioned → Discrepancy; reference severely reduced, unmentioned → Major Error - **Regional dysfunction overcall**: reference normal, report asserts akinesia/dyskinesia or regional RV dysfunction → Discrepancy - **Direction reversal**: reference severely reduced ↔ report "normal/preserved RV function"; reference normal ↔ report "severe RV dysfunction" → Major Error |
| **C3** | **Pulmonary hypertension** | 4 | Normal/Mild/Moderate/Severe PHT (RVSP or TR V_max_-based)^9)^ | - **Implicit normal**: reference normal, unmentioned → Acceptable - **Omission**: reference mild, unmentioned → Discrepancy; reference moderate or severe, unmentioned → Major Error - **Direction reversal**: reference severe PHT ↔ report "normal PA pressure / no PH"; reference normal ↔ report "severe PH" → Major Error |
| **C4** | **IVC and RA pressure** | 4 | RAP categories: 3/8/15/20 mmHg, classified by IVC diameter and collapsibility (plethora): IVC small/normal + collapsible (>50%) → 3; intermediate → 8; IVC dilated + reduced collapse (<50%) → 15; IVC very dilated (>2.5 cm) + minimal collapse → 20. Adjacent pairs: 3↔8, 8↔15, 15↔20.^9)^ | - **Implicit low RAP**: reference 3 or 8, unmentioned → Acceptable - **Omission**: reference 15, unmentioned → Discrepancy; reference 20, unmentioned → Major Error - **IVC diameter reversal**: reference 15 or 20 (IVC dilated state implied), report asserts "IVC not dilated / IVC normal" → Discrepancy - **Direction reversal**: reference 20 ↔ report "RAP 3 / normal RAP" definitive; reference 3 ↔ report "RAP 20 / markedly elevated RAP" definitive; reference 20 ↔ report "IVC not dilated / IVC normal" definitive → Major Error |
| **Domain D. Aorta, Pericardium, Intracardiac Mass, and Congenital Heart Disease (16 points)** | | | | |
| **D1** | **Aorta** | 4 | Four sub-elements; final score = worst rating across scored sub-elements. (A) Aortic size by segment: normal/ dilatation/aneurysm (thoracic: <4.0/4.0–4.5/ ≥4.5 cm; abdominal: <2.5/2.5–3.0/ ≥3.0 cm). Primary segments (root/SOV, ascending) are always scored; secondary segments (arch, descending, abdominal) are scored only when the reference provides a category or the report mentions the segment. (B) Coarctation of the aorta (CoA: none/present/ indeterminate; trigger-based). (C) Acute aortic syndrome (AAS) location/extent (Type A/Type B/indeterminate; scored only when Step 0 AAS-positive). (D) Aortic intraluminal lesion (none/present/ indeterminate; trigger-based). ^10-12)^ | **(A) Aortic size**   - **Implicit normal:** reference normal in a scored segment, unmentioned → Exact - **Omission:** reference dilatation → Discrepancy; reference aneurysm → Major Error - **Segment mis-localization** (abdominal ↔ thoracic) when management differs → Discrepancy - **Direction reversal:** aneurysm ↔ "normal/no dilation"; normal ↔ "aneurysm" → Major Error   **(B) Coarctation of the aorta (trigger-based)**   - **Mismatch:** present ↔ none → Discrepancy - **Direction reversal:** present ↔ "definitely no CoA"; none ↔ "definite/critical CoA" → Major Error   **(C) Acute aortic syndrome location (Step 0 AAS-positive only)**   - Omission or Type A ↔ Type B reversal → Major Error   **(D) Aortic intraluminal lesion (trigger-based)**   - **Mismatch:** present ↔ none → Discrepancy - **Direction reversal:** reference mobile thrombus ↔ "no thrombus" → Major Error |
| **D2** | **Pericardium** | 4 | Three sub-elements; final score = worst rating. (A) Effusion severity: none/trace / small (<10 mm)/moderate (10–20 mm) / large (≥20 mm). "None" subsumes trace /trivial/physiologic/minimal into a single none/trace category. (B) Tamponade physiology: absent/borderline/present/ indeterminate. (C) Constrictive physiology: absent/borderline/present/ indeterminate. Loculated effusion and effusive-constrictive pericarditis modifiers are recorded but not routinely scored. | **(A) Effusion severity**   - **Implicit none/trace**: reference none/trace, unmentioned → Acceptable - **Omission**: reference small → Discrepancy; reference moderate or large → Major Error - **Direction reversal**: none ↔ "large/massive"; large ↔ "no effusion" → Major Error   **(B) Tamponade physiology**   - **Implicit absent**: reference absent, unmentioned → Acceptable - **Omission**: reference present or borderline, unmentioned → Discrepancy - **Direction reversal:** present ↔ "no tamponade"; absent ↔ "tamponade" → Major Error   **(C) Constrictive physiology**   - **Implicit absent**: reference absent, unmentioned → Acceptable - **Omission**: reference present or borderline, unmentioned → Discrepancy - **Direction reversal:** present ↔ "no constrictive physiology"; absent ↔ "constrictive pericarditis/physiology" → Major Error   **Comprehensive normal assertion**: report explicitly negates effusion or asserts "no significant pericardial abnormality" / "pericardium normal" — when reference is normal across all three sub-elements, missing (B) and (C) mentions are scored Exact |
| **D3** | **Intracardiac mass** | 4 | Three sub-elements; final score = worst rating across scored sub-elements. (A) Type: none/thrombus/vegetation/tumor (or non-thrombotic mass)/indeterminate. (B) Location: LV apex/LV cavity (other) /LA body/LAA/RA body/RAA/RV cavity/valve-related (AV/MV/TV/PV)/ other. (C) Mobility (trigger-based; scored only when report mentions mobility): mobile/non-mobile/ highly mobile/indeterminate. Lamble’s excrescences, valvular strands, and nodular thickening are recorded as incidental (non-mass), not as type=tumor/vegetation/thrombus. | **(A) Type**   - **Implicit none**: reference none, mass unmentioned → Exact - **Incidental non-mass mention**: reference none, report mentions Lamble's excrescences / valvular strands / nodular thickening only → Acceptable - **Omission**: reference present, mass completely unmentioned → Discrepancy - **Type mismatch** (thrombus ↔ vegetation/tumor) → Discrepancy - **Active denial / false-positive major finding:** reference present ↔ "no mass / no thrombus / no vegetation"; reference none ↔ "definite thrombus / vegetation / tumor" → Major Error   **(B) Location**   - **Core mismatch** (LAA thrombus ↔ LV apex thrombus) when management pathway (anticoagulation, TEE) differs → Discrepancy   **(C) Mobility** (trigger-based)   - **Direction reversal:** mobile ↔ "fixed/immobile"; fixed ↔ "highly mobile" → Major Error |
| **D4** | **Simple congenital heart disease** | 2 | Three sub-elements; final score = worst rating across scored sub-elements. (A) Lesion type: none/ASD (secundum, primum, sinus venosus)/VSD/PDA/PFO /≥2 lesions/indeterminate. (B) Shunt direction: L→R/R→L/bidirectional/ indeterminate (trigger-based when reference A ≠ none, or report mentions direction). (C) Hemodynamic significance: hemodynamically significant/insignificant/indeterminate (trigger-based when reference A ≠ none, or report mentions significance). ASD–PFO grey-zone tagged when imaging is ambiguous (small interatrial communication). | **(A) Lesion type**   - **Implicit none**: reference none, no shunt/CHD expression → Exact - **ASD–PFO grey-zone tolerance**: reference ASD or PFO ↔ report ASD–PFO range (no definitive trigger) → Acceptable - **Omission**: reference present, shunt/CHD unmentioned → Discrepancy - **Type mismatch** (within simple CHD categories) → Discrepancy - **Active denial / false-positive major finding** (reference present ↔ "no shunt / no ASD/VSD/PDA/PFO / no septal abnormality"; reference none ↔ "definite ASD/VSD/PDA", excluding PFO) → Major Error   **(B) Shunt direction** (trigger-based)   - **Under-spec, L→R**: reference L→R, unmentioned → Acceptable - **Omission or reversal** (R→L or bidirectional, unmentioned; or reference R→L/bidirectional ↔ report "L→R only") → Discrepancy   **(C) Hemodynamic significance** (trigger-based)   - **Under-spec, insignificant**: reference insignificant, unmentioned → Acceptable - **Omission**: reference significant, unmentioned → Discrepancy - **Closure-indicated reversal** (reference insignificant ↔ report "closure indicated") → Major Error |
| **D5** | **Complex congenital heart disease** | 2 | Three sub-elements; final score = worst rating. (A) Presence: none/present/ indeterminate. (B) Category (when present): DORV/Fontan physiology/ interrupted aortic arch/pulmonary atresia /single-ventricle anatomy/ccTGA (L-TGA)/d-TGA after atrial switch (Mustard/Senning)/d-TGA after Rastelli /truncus arteriosus/unrepaired or partially palliated cyanotic CHD. (C) Shunt/flow direction (trigger-based; scored only when report mentions direction): predominant L→R/ predominant R→L/bidirectional/mixing/ indeterminate.^13)^ |  |

The Step 1 Precision Assessment evaluates 25 items grouped into four anatomical domains (left heart structure and function, valves, right heart and pulmonary artery, and aorta/pericardium/intracardiac mass/congenital heart disease). Each item is graded on a five-tier scoring scale (Exact, Acceptable, Discrepancy, Major Error, Not Applicable) weighted by item-specific weights (W); operational rules — including the default adjacent-grade rule, Implicit Normal handling, Indeterminate handling, internal-inconsistency rule, N/A categories, and the cardiomyopathy phenotype trigger — are defined in Supplementary Table S2. The Precision Score is the weighted sum across evaluable items, expressed on a 0–80 scale. Reference categories and grading thresholds reflect the current framework version, anchored to clinical guidelines listed in the Methods, and may be revised as evidence and guidelines evolve. Detailed item-level lexicon mapping (e.g., negation handling, range expressions, abbreviation normalization) and edge-case logic are specified in the master EchoPeer protocol.

Abbreviations: AAS, acute aortic syndrome; AR, aortic regurgitation; ARVC, arrhythmogenic right ventricular cardiomyopathy; AS, aortic stenosis; ASD, atrial septal defect; AV, aortic valve; ccTGA, congenitally corrected transposition of the great arteries; CHD, congenital heart disease; CMP, cardiomyopathy; CoA, coarctation of the aorta; DCM, dilated cardiomyopathy; DORV, double-outlet right ventricle; d-TGA, dextro-transposition of the great arteries; FAC, fractional area change; HCM, hypertrophic cardiomyopathy; heart transplantation; IVC, inferior vena cava; LA, left atrium; LAD, left anterior descending; LAVI, left atrial volume index; LCX, left circumflex; LFLG, low-flow low-gradient; LV, left ventricle; LVAD, left ventricular assist device; LVEDVi, left ventricular end-diastolic volume index; LVEF, left ventricular ejection fraction; left ventricular hypertrophy; LVMi, left ventricular mass index; LVVi, left ventricular volume index; MAC, mitral annular calcification; MR, mitral regurgitation; MS, mitral stenosis; MV, mitral valve; N/A, not applicable; NDLVC, non-dilated left ventricular cardiomyopathy; PA, pulmonary artery; PAU, penetrating atherosclerotic ulcer; PDA, patent ductus arteriosus; PFO, patent foramen ovale; PH, pulmonary hypertension; PV, pulmonary valve; PVL, paravalvular leak; RA, right atrium; RAA, right atrial appendage; RAP, right atrial pressure; RCA, right coronary artery; RCM, restrictive cardiomyopathy; RV, right ventricle; RVEF, right ventricular ejection fraction; RVH, right ventricular hypertrophy; RVSP, right ventricular systolic pressure; RWMA, regional wall motion abnormality; RWT, relative wall thickness; SAVR, surgical aortic valve replacement; S′, tricuspid annular peak systolic velocity; SOV, sinus of Valsalva; TAPSE, tricuspid annular plane systolic excursion; TAV, tricuspid aortic valve; TAVR, transcatheter aortic valve replacement; TEE, transesophageal echocardiography; TEER, transcatheter edge-to-edge repair; TR, tricuspid regurgitation; TRVARC, Tricuspid Valve Academic Research Consortium; TV, tricuspid valve; VSD, ventricular septal defect; W, item weight.

**Supplementary Table S4. Step 0 Failure Rates by Critical-Finding Category and Training Level**

| **Critical-finding category** | **No. of cases** | **Level 1 (n=3)** | **Level 2 (n=5)** | **Level 3 (n=3)** |
| --- | --- | --- | --- | --- |
| Cardiac tamponade | 2 | 33% | 20% | 0% |
| Acute aortic syndrome (AAS) | 1 | 33% | 20% | 0% |
| Acute pulmonary embolism (PE) | 1 | 67% | 60% | 0% |
| Severe valvular obstruction (including LFLG AS, myxoma-induced severe MS, prosthetic valve stenosis) | 5 | 40% | 32% | 13% |

Failure rate = number of failed participant–case combinations / total possible participant–case combinations in that Level (n cases × n participants in Level). Critical-finding categories not represented in the 30-case set (mechanical complication of myocardial infarction, thrombus in transit, LVAD or VA-ECMO complication) are omitted.

**Supplementary Appendix S1. Self-Assessment Questionnaire**

***Self-Assessment Questionnaire for Echocardiography Competency and Experience (COCATS 4–Based)***

Name: _____________________

*Instructions: For checkbox items, mark the applicable option with an “X” or “✓”. For numeric items, enter the appropriate number or check the corresponding range.*

**1. Basic Information**

**Current training year:** ☐ 1st year	☐ 2nd year	☐ ≥3rd year	☐ Other: _____

**Cumulative echocardiography rotation duration:** _____ months

**At your institution, who primarily performs transthoracic echocardiography (TTE)?**

☐ Physician-performed (fellows/physicians acquire images directly)

☐ Sonographer-performed (dedicated sonographers acquire images)

*→ Note: physician-performed TTE counts may be relatively lower in sonographer-based laboratories*

**2. Case Volume (Quantitative Experience)**

**Cumulative TTE studies personally performed (approximate count):**

☐ 0–24	☐ 25–74	☐ 75–149	☐ 150–299	☐ ≥300

**Cumulative TTE studies interpreted or reported (or supervised interpretation):**

☐ 0–49	☐ 50–149	☐ 150–299	☐ 300–749	☐ ≥750

**Experience in additional modalities (enter approximate count; enter 0 if none):**

| **Modality** | **Approximate count** |
| --- | --- |
| TEE observation/participation | _____ studies |
| TEE esophageal intubation (personally performed) | _____ intubations |
| Stress echocardiography observation/participation | _____ studies |
| Contrast echocardiography (including agitated saline/microbubble) | _____ studies |
| Three-dimensional echocardiography (including acquisition/post-processing) | _____ studies |
| Strain imaging / global longitudinal strain (acquisition or interpretation) | _____ studies |

**3. Self-Rated Competency (1–5 scale)**

*Rating scale: 1 = not confident at all; 2 = limited; 3 = moderate; 4 = generally capable; 5 = independently capable*

| **Competency domain** | **Self-rating** |
| --- | --- |
| Standard TTE image acquisition (essential views, Doppler) | ☐ 1 ☐ 2 ☐ 3 ☐ 4 ☐ 5 |
| Assessment of LV systolic function (LVEF) and regional wall motion | ☐ 1 ☐ 2 ☐ 3 ☐ 4 ☐ 5 |
| Diastolic function assessment and grading | ☐ 1 ☐ 2 ☐ 3 ☐ 4 ☐ 5 |
| Integrated interpretation of valvular disease severity (AS/AR/MS/MR/TR) combining quantitative and qualitative measures | ☐ 1 ☐ 2 ☐ 3 ☐ 4 ☐ 5 |
| Report writing (consistent integration of key conclusions, supporting evidence, and limitations) | ☐ 1 ☐ 2 ☐ 3 ☐ 4 ☐ 5 |

**(Optional) Closest match to your current level of independent interpretation:**

☐ Not yet capable (requires full supervision)

☐ Capable for simple cases; complex cases require supervision

☐ Capable for independent interpretation of most cases

**4. Self-Estimated Training Level (Based on COCATS Concept)**

*Please select the option that best describes your current level.*

☐ Level I: Basic performance and interpretation at an introductory stage; independent interpretation not yet possible

☐ Level II: Capable of independent interpretation of echocardiograms, including both routine and complex cases

☐ Level III: Advanced competency, including specialized echocardiographic procedures and capability for teaching, research, or echocardiography laboratory leadership

This questionnaire was administered to all pilot study participants. Level assignment was determined by integrating the self-reported quantitative experience (Section 2), self-rated competency (Section 3), self-estimated training level (Section 4), and supervisor evaluation, with reference to COCATS 4 training thresholds.

**References**

1) Lang RM, Badano LP, Mor-Avi V, et al. Recommendations for cardiac chamber quantification by echocardiography in adults: an update from the American Society of Echocardiography and the European Association of Cardiovascular Imaging. J Am Soc Echocardiogr 2015;28:1-39 e14.

2) Marwick TH, Gillebert TC, Aurigemma G, et al. Recommendations on the Use of Echocardiography in Adult Hypertension: A Report from the European Association of Cardiovascular Imaging (EACVI) and the American Society of Echocardiography (ASE). J Am Soc Echocardiogr 2015;28:727-54.

3) Arbelo E, Protonotarios A, Gimeno JR, et al. 2023 ESC Guidelines for the management of cardiomyopathies. Eur Heart J 2023;44:3503-626.

4) Nagueh SF, Sanborn DY, Oh JK, et al. Recommendations for the Evaluation of Left Ventricular Diastolic Function by Echocardiography and for Heart Failure With Preserved Ejection Fraction Diagnosis: An Update From the American Society of Echocardiography. J Am Soc Echocardiogr 2025;38:537-69.

5) Otto CM, Nishimura RA, Bonow RO, et al. 2020 ACC/AHA Guideline for the Management of Patients With Valvular Heart Disease: A Report of the American College of Cardiology/American Heart Association Joint Committee on Clinical Practice Guidelines. Circulation 2021;143:e72-e227.

6) Pibarot P, Dumesnil JG. Aortic stenosis suspected to be severe despite low gradients. Circ Cardiovasc Imaging 2014;7:545-51.

7) Pandian NG, Kim JK, Arias-Godinez JA, et al. Recommendations for the Use of Echocardiography in the Evaluation of Rheumatic Heart Disease: A Report from the American Society of Echocardiography. J Am Soc Echocardiogr 2023;36:3-28.

8) Hahn RT, Lawlor MK, Davidson CJ, et al. Tricuspid Valve Academic Research Consortium Definitions for Tricuspid Regurgitation and Trial Endpoints. J Am Coll Cardiol 2023;82:1711-35.

9) Mukherjee M, Rudski LG, Addetia K, et al. Guidelines for the Echocardiographic Assessment of the Right Heart in Adults and Special Considerations in Pulmonary Hypertension: Recommendations from the American Society of Echocardiography. J Am Soc Echocardiogr 2025;38:141-86.

10) Isselbacher EM, Preventza O, Hamilton Black J 3rd, et al. 2022 ACC/AHA Guideline for the Diagnosis and Management of Aortic Disease: A Report of the American Heart Association/American College of Cardiology Joint Committee on Clinical Practice Guidelines. Circulation 2022;146:e334-e482.

11) Czerny M, Grabenwoger M, Berger T, et al. EACTS/STS Guidelines for diagnosing and treating acute and chronic syndromes of the aortic organ. Eur J Cardiothorac Surg 2024;65:ezad426.

12) Obel LM, Diederichsen AC, Steffensen FH, et al. Population-Based Risk Factors for Ascending, Arch, Descending, and Abdominal Aortic Dilations for 60-74-Year-Old Individuals. J Am Coll Cardiol 2021;78:201-11.

13) Gurvitz M, Krieger EV, Fuller S, et al. 2025 ACC/AHA/HRS/ISACHD/SCAI Guideline for the Management of Adults With Congenital Heart Disease: A Report of the American College of Cardiology/American Heart Association Joint Committee on Clinical Practice Guidelines. J Am Coll Cardiol 2026;87:822-976.

14) Taub CC, Stainback RF, Abraham T, et al. Guidelines for the Standardization of Adult Echocardiography Reporting: Recommendations From the American Society of Echocardiography. J Am Soc Echocardiogr 2025;38:735-74.
